# Salting-out assisted liquid–liquid extraction combined with LC–MS/MS for the simultaneous determination of seven organic UV filters in environmental water samples: method development and application

**DOI:** 10.1007/s11356-023-29646-8

**Published:** 2023-09-15

**Authors:** Megan Carve, Navneet Singh, Matthew Askeland, Graeme Allinson, Jeff Shimeta

**Affiliations:** 1 School of Science, RMIT University, Melbourne, VIC Australia; 2 ADE Consulting Group, Williamstown North, VIC 3016 Australia

**Keywords:** UHPLC–MS/MS, SALLE, Benzophenone, Avobenzone, Ethylhexyl-methoxycinnamate, Sunscreen, UV stabilisers

## Abstract

Organic UV filters (OUVFs), the active ingredient in sunscreens, are of environmental concern due to reported ecotoxicological effects in aquatic biota. Determining the environmental concentrations of these chemicals is essential for understanding their fate and potential environmental risk. Salting‐out assisted liquid–liquid extraction (SALLE) coupled with liquid-chromatography tandem mass spectrometry (LC–MS/MS) was developed for simultaneous extraction, separation, and quantification of seven OUVFs (2,4-dihydroxybenzophenone, 2,2′,4,4′-tetrahydroxybenzophenone, 4-methylbenzylidene camphor, butyl-methoxy-dibenzoyl methane, octocrylene, octyl methoxycinnamate, and oxybenzone). Method detection limits (MDLs) ranged from 11 to 45 ng/L and practical quantification limits (PQLs) from 33 to 135 ng/L. Method trueness, evaluated in terms of recovery, was 69–127%. Inter-day and intra-day variability was < 6% RSD. The coefficients of determination were > 0.97. The method was applied to river and seawater samples collected at 19 sites in and near Port Phillip Bay, Australia, and temporal variation in OUVF concentrations was studied at two sites. Concentrations of OUVF were detected at 10 sites; concentrations of individual OUVFs were 51–7968 ng/L, and the maximum total OUVF concentration detected at a site was 8431 ng/L. Recreational activity and water residence time at the site contributed to OUVF’s environmental presence and persistence. The benefits of the SALLE-LC–MS/MS method include its simple operation, good selectivity, precision over a wide linear range, and that obtained extracts can be directly injected into the LC–MS/MS, overall making it an attractive method for the determination of these OUVFs in environmental water matrices. To our knowledge, this is the first report of the occurrence of OUVFs in Port Phillip Bay, Australia.

**Supplementary Information:**

The online version contains supplementary material available at 10.1007/s11356-023-29646-8.

## Introduction

Organic UV filters (OUVFs) are synthetic compounds that protect against damage from ultraviolet radiation (Ramos et al. 2015). They are the active ingredient in sunscreens and are incorporated in a wide range of personal care and manufactured products (e.g. shampoos, cosmetics, plastics, textiles, paints). Due to their wide use, they can enter freshwater and marine environments directly, for example when people engage in recreational activities or via industrial and wastewater discharge (Benedé et al. 2014; Tsui et al. 2014; Labille et al. 2020; O’Malley et al. 2020). Organic UV filters are becoming an important class of contaminants of emerging concern as they are increasingly detected in the environment. Toxicological data regarding OUVFs has shown harmful effects in aquatic species related to their endocrine-disrupting potential and genotoxic capabilities (Schlumpf et al. 2004; Downs et al. 2014, 2016; Wang et al. 2016; Ozaez et al. 2016; Carve et al. 2021). Consequently, the sale of sunscreens containing certain OUVFs, such as oxybenzone (BP-3) and octocrylene (OCT), have been restricted in some regions (Republic of Palau 2018; Hawaii 2018; Miller et al. 2021). Determining the environmental concentrations of these contaminants is essential for understanding their fate and potential risk to aquatic environments.

The physicochemical properties of OUVFs determine their fate in the environment and are an important consideration for optimization of analytical detection methods (Cadena-Aizaga et al. 2020). Typically, OUVFs contain single or multiple aromatic structures attached to hydrophobic groups (Ramos et al. 2016). Most are lipophilic, non-volatile, have a log K_ow_ ≥ 4, and low to nil aqueous solubility. OUVFs have a limited absorption band spectrum; thus, sunscreens and other commodities often contain multiple OUVFs to provide protection against UVA (320–400 nm) and UVB (280–320 nm) (Ramos et al. 2016). Consequently, mixtures of OUVFs are expected to occur and have been detected at ng–mg/L levels in water samples from coastal and freshwater environments (Benedé et al. 2014; Tsui et al. 2014; Allinson et al. 2018; Labille et al. 2020; O’Malley et al. 2020). Oxybenzone (benzophenone-3; BP3) is the most frequently detected OUVF (Cadena-Aizaga et al. 2020); other more commonly observed OUVFs are 4-methylbenzylidene camphor (4-MBC), octyl methoxycinnamate (OMC), and octocrylene (OCT) (Cuccaro et al. 2022).

Determining the environmental concentrations of OUVFs is challenging due to the low concentrations and complex matrices typical of environmental water samples (Cadena-Aizaga et al. 2020). Accurate determination relies on high sensitivity of the analytical method and optimization of the sample preparation method (extraction, purification, and concentration). Extraction techniques more used include liquid–liquid extraction (LLE) (Jeon et al. 2006; Pintado-herrera et al. 2016), stir-bar sorptive extraction (Kawaguchi et al. 2006; Pintado-Herrera et al. 2013), single-drop microextraction (SDME) (Okanouchi et al. 2008) dispersive liquid microextraction (Tarazona et al. 2010), and solid-phase extraction (SPE) (Negreira et al. 2009; León et al. 2010; Kameda et al. 2011), with SPE being the most popular (Gago-Ferrero et al. 2013; Ramos et al. 2015). Disadvantages of some of these approaches may include large sample (~ 1 L) and solvent volume, lengthy extraction times, or the inadequacy of the method for the extraction of polar analytes (Cadena-Aizaga et al. 2020).

Salting-out assisted liquid–liquid extraction (SALLE) is based on LLE and has several advantages over other extraction methods. In SALLE, a salt is added to achieve the separation of aqueous phase from the partially miscible organic phase, and simultaneously, the target analytes are extracted into the separated organic phase (Valente et al. 2013; Wen et al. 2013). The organic phase can then be used directly for analysis. SALLE is a relatively simple method to perform and requires a small volume of samples and solvents compared to the other commonly used extraction techniques (e.g. SPE) (Razmara et al. 2011). SALLE has been successfully applied for the determination of a range of chemicals including pesticides and synthetic dyes in different types of matrices (Razmara et al. 2011; Valente et al. 2013; Wen et al. 2013; Gure et al. 2014).

In this study, SALLE was optimised for the extraction of seven OUVFs from environmental water samples. The target analytes were 2,4-dihydroxybenzophenone (BP-1), 2,2′,4,4′-tetrahydroxybenzophenone (BP-2), oxybenzone (BP-3), 4-methylbenzylidene camphor (4-MBC), butyl-methoxy-dibenzoylmethane (B-MDM), octyl methoxycinnamate (OMC), and octocrylene (OCT). The approach was validated by evaluating method detection limits (MDLs), practical quantification limits (PQLs), method trueness and inter-day precision, method linearity, and matrix recovery. The method was used to determine the concentrations of the target analytes in seawater and river water samples collected at 19 sites in and near Port Phillip Bay, Australia. To our knowledge, this is the first report of OUVF concentrations in Port Phillip Bay.

## Materials and methods

### Chemicals and standards

Analytical standards and the isotopically labelled analogue oxybenzone-(phenyl-^13^C_6_) were purchased from Novachem (Heidelberg West, VIC, Australia). The target analytes and their physical–chemical properties are shown in Table 1. Standards were purchased as solutions of 100 µg/mL in methanol (4-MBC, B-MDM, OMC) or acetonitrile (ACN; BP-3, OCT), except for BP-2, which was not dissolved in solvent, and BP-1 which was a 1000 µg/mL solution in 8:2 hexane:acetone (v/v). Linear calibration curves were constructed by gravimetric dilution of standard stock solutions in 70:30 ACN:ultrapure water (> 18Ω, Milli-Q, Millipore). All solvents and chemicals were of analytical grade. Formic acid, glacial acetic acid, and ammonium acetate (≥ 99.99%) were purchased from SigmaAldrich (Castle Hill, NSW, Australia). A combined standard solution (100 µg/L) and a surrogate stock solution (50 µg/L oxybenzone-(phenyl-^13^C_6_)) used for spiking were prepared in ACN (LC–MS grade, Honeywell, USA, and LiChrosolv hypergrade, Merck Millipore, Australia) and stored at − 20 °C. Sodium chloride (NaCl, ≥ 99.0%), sodium sulphate (Na_2_SO_4_, ≥ 99.0%), calcium chloride (CaCl, ≥ 99.0%), and magnesium chloride (MgSO_4_, ≥ 99.0%) were purchased from SigmaAldrich (Castle Hill, NSW, Australia) and Rowe Scientific (Dovetone, VIC, Australia). Quality controls (QCs), matrix spike (seawater spiked with OUVF standard and isotopically labelled surrogate), blanks spike (ultrapure water spiked with OUVF standard and isotopically labelled surrogate), and blank (ultrapure water) were prepared on the day of analysis and analysed alongside environmental samples. All QCs were extracted using the SALLE protocol described below.

**Table 1:** The properties of the target organic UV filters including their molecular weight (MW), octanol–water partition coefficient (K_ow_), and soil adsorption coefficient (K_oc_)

Names	Abbreviation	CAS #	Hill formula	MW	pKa	Log K_ow_	K_oc_
2,4-dihydroxybenzophenone	BP-1	131–56-6	C13H10O3	214.22	7.09^b^	3.48^b^	2885^c^
2,2′,4,4′-tetrahydroxybenzophenone	BP-2	131–55-5	C13H10O5	246.218	6.75^b^	3.52^b^	7726^c^
Oxybenzone	BP-3	131–57-7	C14H12O3	228.247	3.79^a^	3.79^a^	1268^c^
4-methylbenzylidene camphor	4-MBC	36861–47-9	C18H22O	254.37	-^d^	4.95^a^	12,210^c^
Butyl-methoxy-dibenzoylmethane	B-MDM	70356–09-1	C20H22O3	310.393	9.74^a^	4.51^a^	1705^c^
Octocrylene	OCT	6197–30-4	C24H27NO2	361.485	-^d^	6.88^a^	411,300^c^
Octyl methoxycinnamate	OMC	5466–77-3	C18H26O3	290.403	-^d^	5.80^a^	12,280^c^

^a^Available in Cadena–Aizaga et al. (2020)

^b^Predicted data from ChemAxon accessed via: https://hmdb.ca/

^c^Predicted data generated using the US Environmental Protection Agency’s EPISuite accessed via: www.chemspider.com

^d^Data not found

### Optimization of SALLE procedure

Each seawater sample (10 mL) spiked with combined standard solution was mixed with acidified ACN (0.5 M Formic acid) at a ratio of 1:1 (v/v) in analytically certified amber glass vials. Samples were vortexed for 30 s and then secured in a TCLP Rotary Agitator and rotated for 1 h at 30 ± 2 rpm at ambient temperature. To initiate the salting-out process, 4 or 8 g of either NaCl, Na_2_SO_4_, CaCl, or MgCl was added to each vial. Vials were vortexed for 1 min to achieve two distinct and well-separated phases. An aliquot from the ACN layer was filtered (0.22 µm cellulose filters, Terumo Australia pty ltd, Macquarie Park, NSW), and 500 µl was transferred to a LC vial to which 500 µl of acidified (0.5 M Formic acid) ultrapure water was added in preparation for chromatographic analysis. All the experiments were performed at room temperature (20 °C), and samples were protected from light to avoid possible photodegradation.

### Instrumentation and chromatographic analysis

LC–MS/MS analysis was performed using the combined Shimadzu Nexera Z2 UHPLC and LCMS-8060 system equipped with an electrospray ionisation source and coupled with the Nexera X2 SIL-30ACMP Autosampler (Shimadzu). System control was with LabSolutions LCMS software (Shimadzu). Chromatographic separations were performed on a C18 column (Shim-pack XR-ODS, 3 mm I.D. × 30 mm, 1.62 µm, Shimadzu) with a guard column (Shim-pack XR-ODS, 3 mm I.D. × 30 mm, Shimadzu). Column temperature was 40 °C, and the injection volume was 40 µL. Mobile phases A and B were ACN, and a mixture of 5 mM ammonium acetate and 0.05% acetic acid in ultrapure water (v/v), respectively. The gradient was programmed as follows: 0.0–1.0 min, 10% A; 1.0–2.0 min, 50% A; 2.0–6.0 min, 100% A; 6.0–10.0 min, 100% A; and back to 10% in 1.0 min. The total time of analysis was 13.5 min. Oxybenzone-(phenyl-^13^C_6_) was used as a surrogate. Tandem mass spectrometer (MS/MS) conditions were optimised for each OUVF by varying MS/MS parameters using injections of analytical standard. General parameters were as follows: interface temperature 300 °C, DL temperature 250 °C, heat block temperature is 400 °C, drying gas flow is 10 L/min, heating gas flow 10 L/min, and nebulizing gas flow is 3 L/min. Electrospray ionisation in both negative and positive ion modes was performed in multiple reaction monitoring (MRM) conditions. The optimised MRM transitions are summarised in Table 2.

**Table 2:** Analytical parameters used in this study retention times and optimized ESI–MS/MS conditions for the organic UV filters (OUVFs) oxybenzone (BP-3), butyl-methoxy-dibenzoylmethane (B-MDM), octyl methoxycinnamate (OMC), 4-methylbenzylidene camphor (4-MBC), 2,2′,4,4′-tetrahydroxybenzophenone (BP-2), octocrylene (OCT), and 2,4-dihydroxybenzophenone (BP-1)

OUVF	Rt^a^ (min)	Acquisition mode	Precursor (m/z)	Product 1 (m/z)	Product 1 RCE^b^ (%)	Product 2 (m/z)	Product 2 RCE^b^ (%)	Product 3 (m/z)	Product 3 RCE^b^ (%)
BP-1	4.773	Negative	212.99	91.00	15.00	135.05	11.11	169.10	11.11
BP-2	4.047	Negative	245.00	135.05	8.33	109.05	12.22	91.0	15.00
BP-3	5.726	Negative	226.99	211.20	12.78	215.15	10.00	NA	NA
4-MBC	6.697	Positive	255.50	178.10	21.67	119.2	12.22	180.1	15.00
B-MDM	6.793	Positive	311.00	161.10	12.22	135.10	12.78	77.15	27.78
OMC	7.296	Positive	291.00	161.20	9.44	179.30	5.56	NA	NA
OCT	7.260	Positive	362.30	250.10	6.67	232.00	13.33	204.15	19.44

^a^*Rt* retention time

^b^*RCE* collision energy relative to mass spectrometer maximum available energy of 180 V

### Method evaluation and quantitation limits

The SALLE combined with LC–MS/MS method was validated with respect to method linearity, detection limits (MDLs), practical quantification limits (PQLs), method trueness, and intra-day and inter-day precision. Method linearity was investigated over a concentration range of 0.04–1.5 ng/mL by plotting corresponding concentrations estimated from LC–MS/MS peak areas versus nominal OUVF concentrations and using least squares regression analysis in the R stats package (R Core Team 2020) in R Studio (v 2022.07.1, R v. 4.1.3). For each OUVF, MDLs, and PQLs were calculated using Eqs. 1 and 2, where t is the one-sided *t* distribution (*t* = 2.821), and *I* is the injected concentration (*I* = 200 pt).

1 $$\mathrm{MDL}=\mathrm{t}\times\upsigma \times \mathrm{I }/\mathrm{ mean\;peak\;area}$$

2 $$\mathrm{PQL}=\mathrm{MDL}\times 3$$

Instrument inter-day and intra-day precision was assessed by calculating the relative standard deviation (%RSD) as in Eq. 3, where Xc is the mean surrogate-corrected value (*n* = 7).

3 $$\mathrm{\%RSD}=\left(\upsigma /{\mathrm{x}}_{\mathrm{c}}\right)\times 100$$

Method trueness was assessed by calculating the percent recovery for spiked seawater and Milli-Q water samples as in Eq. 4, where *Sc* is the spiking concentration and *Xc* is the mean surrogate-corrected value (*n* = 2).

4 $$\mathrm{method\;trueness }\left(\mathrm{\%}\right)=\left({\mathrm{X}}_{\mathrm{c}}/\mathrm{Sc}\right)\times 100$$

### Application of SALLE-LC/MS to environmental samples

**Fig. 1:**
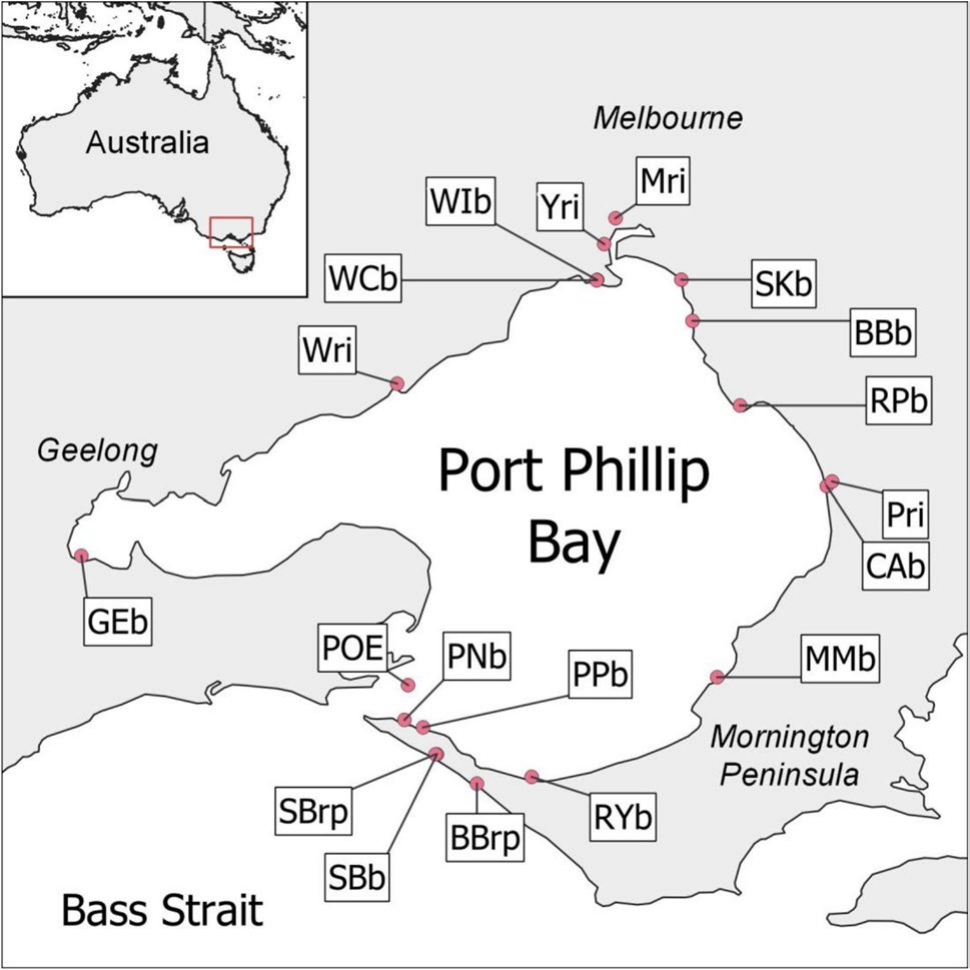
The sampling sites in and around Port Phillip Bay (PPB), Australia. The water types sampled were Rivers (Maribyrnong River, Mri, *n* = 3; Patterson River, Pri, *n* = 3; Werribee River, Wri, *n* = 3); Yarra River, Yri, *n* = 3), PPB beaches (Brighton, BBb, *n* = 3; Carrum, Cab, *n* = 3; Geelong Eastern, Geb, *n* = 3; Mount Martha, MMb, *n* = 3; Portsea Pier, PPb, *n* = 3; Quarantine Station, PNb, *n* = 3; Ricketts Point, RPb, *n* = 3; Rye Pier, RYb, *n* = 3 per time point, 9 samples in total; St Kilda, SKb, *n* = 3; Williamstown, WIb, *n* = 3; Williamstown Crystals, WCb, *n* = 3), Ocean Beaches (Sorrento Ocean beach, SBb, *n* = 3)), and rockpools (Sorrento rockpool, SBrp, *n* = 3 per time point, 9 samples in total; Bridgewater Bay, BBrp, *n* = 3), and one site that was located toward the center of the entry to Port Phillip Bay at Popes Eye (POE, *n* = 3)

Environmental water samples were collected at 19 sites in and around Port Phillip Bay (PPB), Australia (*n* = 3 per site and sampling time) (Fig. 1). Four sites were rivers terminating in PPB (*n* = 12), 11 sites were beaches in PPB that are popular for recreational activities (*n* = 39), 3 sites were at Mornington Peninsula Ocean beaches (beach site *n* = 9, and rock pools *n* = 12), and 1 site was located near the centre of the entrance to PPB (Popes Eye, *n* = 3). To investigate temporal variation, samples were collected at two sites (Rye Bay Beach and Sorrento rockpool) in the early morning, midday, and late evening (*n* = 3 per site, per time point).

Samples were collected by opening an analytically certified amber glass vial (40 mL) under the water’s surface, allowing the bottle to passively fill, and replacing the lid while the vial was still submerged. Samples were collected 10 to 20 cm under the water’s surface and 3 to 10 m from shore. Due to the risk of sample contamination, the use of personal care products containing OUVFs was avoided before and during the sampling. At each site, the number of people engaged in recreational activity was estimated by counting the number of beachgoers in the water. Climate data on daily solar exposure (MJ m^−2^) and weather parameters (air and water temperature, wind speed and direction, tidal movement) were measured during sampling or compiled from publicly available data on the Australian Bureau of Meteorology website (2022) (Supplementary file 1, Table S1). Samples were stored in the dark on ice during collection and transported to ADE Consulting laboratory, Melbourne, where they remained in storage in the dark at 4 °C until analysis.

## Results and discussion

### Optimization of SALLE

Sample preparation is integral to the sensitivity and trueness of an analytical method (Płotka-Wasylka et al. 2021). In SALLE, important factors that influence performance are the selected salt and salt concentration and the selected organic solvent (Camino-Sánchez et al. 2011). The salt’s anion is considered responsible for efficient phase separation, and different salt types and salt concentrations can induce varying grades of phase separation due to differing ionic strengths of the aqueous donor phase, which affects the analytes solubility in the aqueous phase and consequent transfer to the organic phase (Asensio-ramos et al. 2011; Wen et al. 2013; Benedé et al. 2014; Gure et al. 2014). The salting-out effect can improve the extraction of polar analytes from the aqueous phase, particularly for extraction of polar compounds (log Kow < 4) with hydroxyl groups (e.g. BP-3) (Pintado-Herrera et al. 2014; Vila et al. 2016a).

**Fig. 2:**
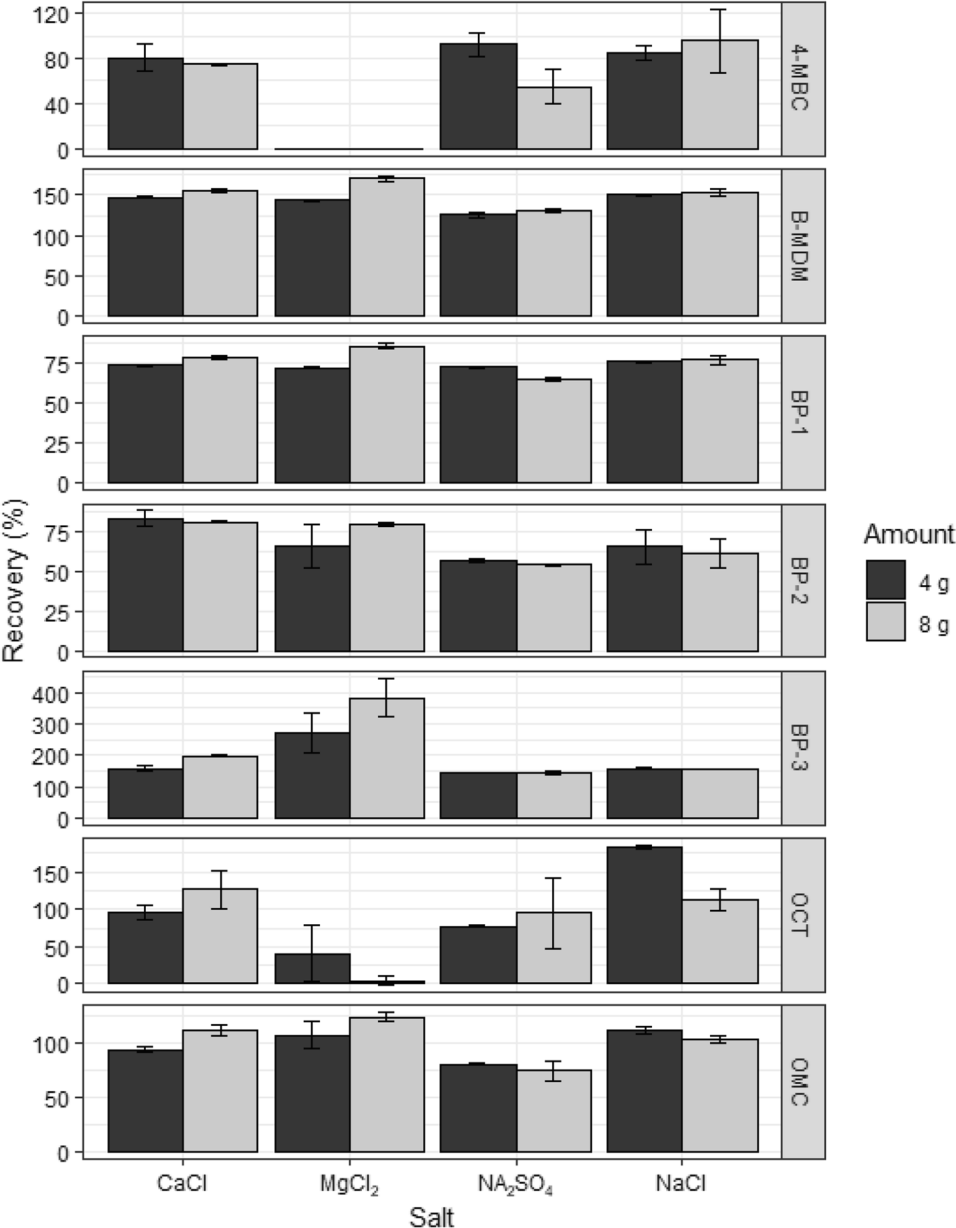
The effect of different salts (NaCl, Na_2_SO_4_, CaCl, and MgCl) and two salt amounts (4 and 8 g) on extraction efficiency (mean ± standard error) of seven organic UV filters from seawater using SALLE. Extraction conditions: 10 mL sample volume, 10 mL acetonitrile. Target analytes were 4-methylbenzylidene camphor (4-MBC), butyl-methoxy-dibenzoylmethane (B-MDM), 2,4-dihydroxybenzophenone (BP-1), 2,2′,4,4′-tetrahydroxybenzophenone (BP-2), oxybenzone (BP-3), octocrylene (OCT), and octyl methoxycinnamate (OMC)

Four different salts (NaCl, Na_2_SO_4_, CaCl, MgCl) at two different amounts (4 or 8 g/sample) were tested in this study (Fig. 2). All salts induced phase separation at the chosen concentrations. In most cases, increasing the salt amount from 4 to 8 g provided only a small change in recovery (Fig. 2). For subsequent experiments, Na_2_SO_4_ was chosen since it is relatively non-toxic and produced good phase separation and recovery of target analytes (Table 4). Furthermore, SO_4_^2−^ is ranked above Cl^−^ in the Hofmeister series, thus expected to be more efficient at phase separation than Cl^−^ (Alshishani et al. 2017). For Na_2_SO_4_, the difference in analyte recovery between an addition of 4 g or 8 g of salt was < 5% for BP-2, BP-3, and B-MDM, and < 7% for BP-1 and OMC. For 4-MBC, addition of 4 g Na_2_SO_4_ increased mean recoveries from 55.1 to 92.1%, whereas for OCT, addition of 8 g Na_2_SO_4_ increased mean recoveries from 76.8 to 94.9%. Poor phase separation was observed from an amount of Na_2_SO_4_ < 2.5 g.

Acetonitrile was chosen as the organic solvent due to its attractive properties for use in SALLE, specifically its highly polarity, miscibility in water, relatively low toxicity, and that it can be directly injected into LC system (Gure et al. 2014; Sereshti et al. 2014). Solvent volume is an important consideration that can affect SALLE efficiency: too small a volume results in the phase boundary between acetonitrile and sample being too difficult to distinguish, whereas too large a volume results in over dilution of analytes (Sereshti et al. 2014). A volume of 10 mL was found to be suitable for the application and used in subsequent experiments.

### Analytical performance

**Fig. 3:**
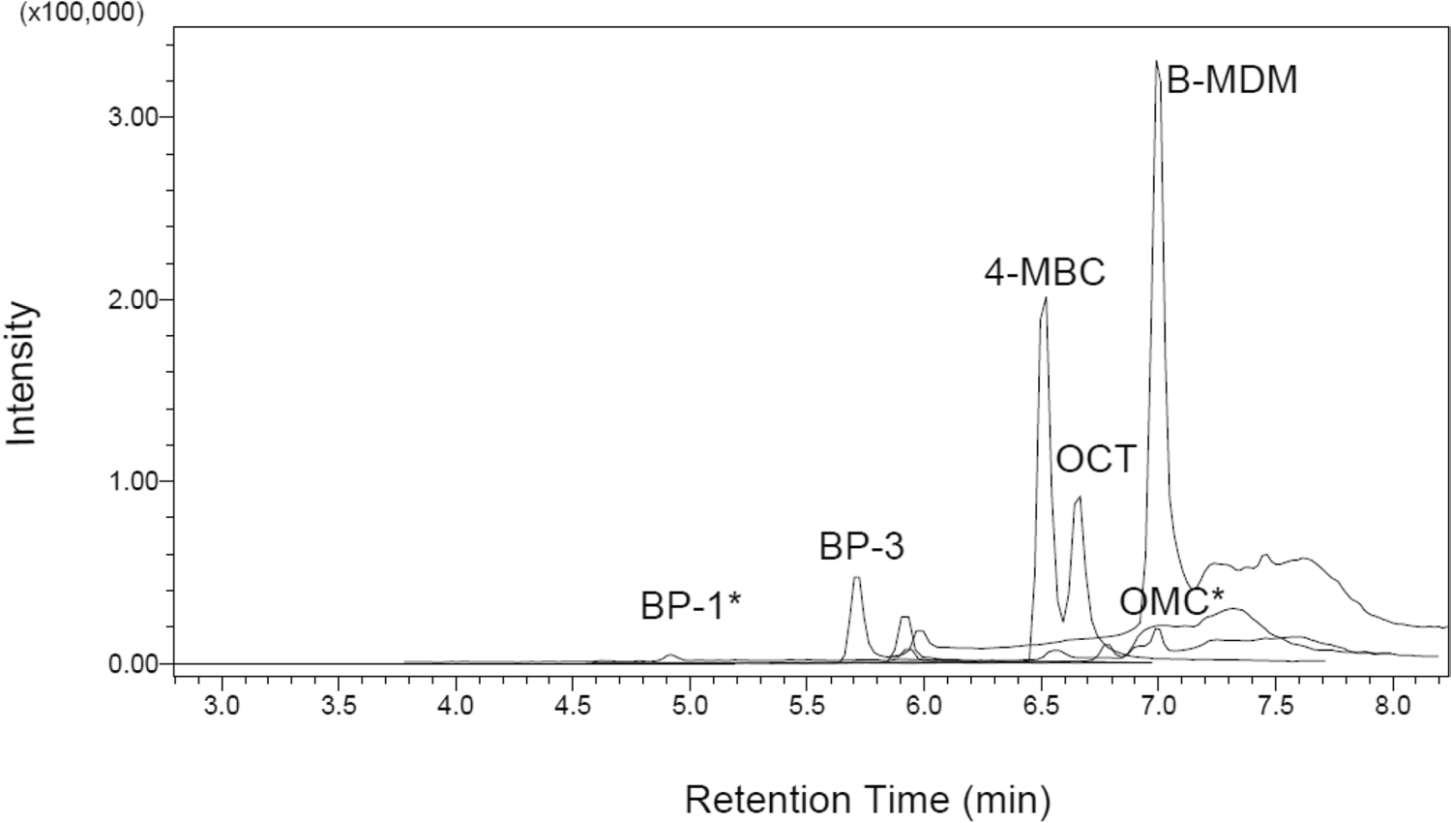
Representative chromatogram for an environmental sample showing organic UV filter mixture: oxybenzone (BP-3), octocrylene (OCT), 4-methylbenzylidene camphor (4-MBC), and butyl-methoxy-dibenzoylmethane (B-MDM), 2,4-dihydroxybenzophenone (BP-1), and octyl methoxycinnamate (asterisk indicates below MDL)

Separation, identification, and quantification of OUVFs were performed using liquid-chromatography tandem–mass spectrometry (LC–MS/MS). Analytes separated along the LC column within 13 min. The addition of formic acid increased the sharpness and resolution of chromatic peaks. Furthermore, addition of formic acid is critical to disrupting compound–protein binding which may occur in environmental samples (Picot Groz et al. 2014). The behaviour of all compounds was linear and exhibited a direct proportional relationship between the amount of each analyte and the chromatographic response. The coefficients of determination (*R*^2^) were above 0.97 (Table 3). Inter-day and intra-day precision, expressed in terms of relative standard deviation (%RSD), was < 6% RSD for all compounds (Table 3). Method detection limits (MDLs) ranged from 11 to 45 ng/L and practical quantification limits (PQLs) from 33 to 135 ng/L (Table 3). Method trueness was assessed in a recovery study carried out with seawater and Milli-Q water samples at two concentrations (Table 4). For Milli-Q samples, average recovery values for the two spike concentrations were 91–109%. Seawater samples also showed good recovery, with average recovery values for the two spike concentrations being 69–127%. A representative chromatogram for an environmental sample is shown in Fig. 3 and for the OUVF combined standards solution is shown in Fig. S1 (Supplementary file 1). A comparison with other methods for determining OUVF concentrations in seawater published in the last 10 years is provided in Table 5. There were some differences among methods regarding the number of targeted OUVFs, analytical performance, and sample volume; however, overall the analytical performance of the methods was similar. All studies had recoveries > 63%. LOD were at trace concentrations in all cases (ng/L). Most methods required a sample volume of ≥ 100 mL, but solid phase microextraction techniques required a smaller sample volume of 10–100 mL. Similar to microextraction techniques, our presently described SALLE method has the advantage of requiring a small sample volume of 10 mL.

**Table 3:** Linear relationship parameters, method detection limits (MDLs), and practical quantification limits (PQLs), inter-day and intra-day precision for target organic UV filters (OUVFs): 2,4-dihydroxybenzophenone (BP-1), 2,24,4-tetrahydroxybenzophenone (BP-2), oxybenzone (BP-3), 4-methylbenzylidene camphor (4-MBC), butyl methoxydibenzoylmethane (B-MDM), octyl methoxycinnamate (OMC), and octocrylene (OCT)

OUVF	Slope (mean ± RSE^**1**^)	Intercept (mean ± RSE)	Multiple ***R***^2^	MDL (ng/L)	PQL (ng/L)	Inter-day precision (RSD %)	Intra-day precision (RSD %)	
							400 ng/L	1000 ng/L
BP-1	1.008 ± 0.009	20.240 ± 9.355	0.9982	11.9	35.7	3.618	2.179	3.189
BP-2	0.980 ± 0.013	27.127 ± 13.479	0.9962	28.2	84.6	3.977	3.196	2.66
BP-3	1.029 ± 0.013	11.197 ± 13.240	0.9967	18.6	55.8	4.425	3.089	2.510
4-MBC	1.070 ± 0.018	− 0.999 ± 17.865	0.9944	45.0	135	4.170	3.090	2.762
B-MDM	1.009 ± 0.015	25.199 ± 15.060	0.9955	11.0	33	5.557	5.062	3.381
OMC	1.069 ± 0.025	− 4.178 ± 25.091	0.9890	18.7	56.1	5.425	5.271	3.625
OCT	0.945 ± 0.031	10.901 ± 25.376	0.9739	16.7	50.1	5.012	5.413^2^	3.723^3^

^1^Residual standard error (*RSE*); ^2^spiking concentration 100 ng/L; ^3^spiking concentration 500 ng/L

**Table 4:** Method trueness assessed by percent recovery for seawater and Milli-Q samples spiked with target organic UV filters (OUVFs): 2,4-dihydroxybenzophenone (BP-1), 2,24,4-tetrahydroxybenzophenone (BP-2), oxybenzone (BP-3), 4-methylbenzylidene camphor (4-MBC), butyl methoxydibenzoylmethane (B-MDM), octyl methoxycinnamate (OMC), and octocrylene (OCT). Values are mean ± standard error

OUVF	Milli-Q recoveries (%)		Seawater recoveries (%)	
	400 ng/L	1000 ng/L	400 ng/L	1000 ng/L
BP-1	101.23 ± 0.83	105.93 ± 1.28	69.35 ± 0.80	114.53 ± 0.57
BP-2	102.70 ± 1.26	98.98 ± 1.20	97.09 ± 2.05	126.66 ± 11.48
BP-3	100.89 ± 1.22	99.34 ± 1.14	86.25 ± 3.35	124.65 ± 0.15
4-MBC	99.06 ± 2.16	108.84 ± 1.34	80.69 ± 12.02	114.00 ± 5.90
B-MDM	98.94 ± 1.98	101.38 ± 1.41	83.65 ± 0.28	112.41 ± 9.71
OMC	98.33 ± 2.25	91.79 ± 1.76	80.81 ± 3.84	117.25 ± 4.81
OCT	92.02 ± 4.98	103.90 ± 6.47	91 ± 0.05	116.20 ± 6.10^1^

^1^100 ng/L spiking concentration

**Table 5:** Comparison with analytical methods applied in the detection of organic UV filters (OUVFs) in seawater published in the last 10 years

Extraction technique ^a^	Sample volume	Extraction conditions	Instrument technique	OUVF ^b^	Recoveries (%)	LOD (ng/L)	LOQ (ng/L)	*R*^2^	RSD (%)	Reference
LLE	200 mL	Hexane (100 mL), 20 min + evaporation time	GC–MS	OMC	90 ± 3.7	0.082	0.27	NR^*^	NR	Sankoda et al. (2015)
				OD-PABA	86 ± 1.8	0.096	0.32			
				4-MBC	89 ± 0.7	0.15	0.5			
				EHS	120 ± 3.3	0.099	0.33			
	500 mL	n-octanol (1 mL), 35 min	LC–MS/MS	BP	88.6–106.8	14.5	48.3	0.9938	10.1	Kung et al. (2018)
				BP-1	94.4–103.5	12.5	41.6	0.9982	5.0	
				BP-3	86.2–109.3	10.3	34.4	0.9991	12.3	
				BP-8	96.6–103.5	9.5	31.6	0.9981	3.5	
				4-MBC	95.0–109.7	10.9	36.4	0.9989	8.1	
DLLME	5 mL	pH-adjusted to 2.5 with glacial acetic acid, acetone (250 µL) and chloroform (50 µL)	GC–MS	EHS	112–117	26	85	0.9991	5–11.3	Benedé et al. (2014)
				HMS	88–97	14	46	0.994	5.5–12.1	
				IAMC	97–107	23	78	0.998	4.1–11.3	
				4-MBC	82–88	10	33	0.996	2.2–12.7	
				BP-3	111–114	30	899	0.997	6.2–13.9	
				OMC	87–99	14	47	0.998	3.1–9.9	
				EDP	90–95	29	98	0.997	6.7–12.3	
				OCT	91–104	27	91	0.994	8.2–14.1	
SBDLME	25 mL	25 mL magnetic ionic liquid [P^+^_6,6,6,14_] [Ni(hfacac)_3_^−^], adjusted to pH 4 with sodium dihydrogen phosphate ≤ 0.1 M and ortho-phosphoric acid, 5% NaCl, stirred for 10 min	GC–MS	EHS	114–117	9.9	32.5	0.999	1.4–14.0	Chisvert et al. (2017)
				HMS	102–104	11.3	37.3	0.999	3.7–14.7	
				IAMC	109–113	13.1	43.1	0.998	5.5–12.0	
				4-MBC	97–102	15.2	50.2	0.997	4.8–13.3	
				BP-3	91–95	10.4	34.3	0.996	2.8–11.2	
				OMC	91–95	15.3	50.5	0.997	3.2–10.5	
				EDP	110–112	26.7	88.2	0.993	1.4–13.2	
				OCT	95–103	21.2	69.9	0.995	5.3–11.5	
SALLE	50 mL	Filtered, adjusted to pH3, methyl tert-butyl ether (5 mL), 10 g Na_2_SO_4_ + 2 min + evaporation time	LC–MS/MS	BP-8	89	8	20	0.992	8–16	Labille et al. (2020)
				BP-3	93	1	5	0.997		
				OCT	117	5	15	0.995		
				B-MDM	97	5	15	0.998		
				OMC	102	5	15	0.998		
SPE	350 mL	OASIS HLB, 27 mL 50:50 v/v methanol: ethyl acetate, 3.5 mL of 5% (w/v) Na2EDTA + evaporation time	HPLC–ESI–MS/MS	OD-PABA	73 ± 4	0.03	NR	0.9927	1.5–7.9	Tsui et al. (2014)
				4-MBC	83 ± 4	0.28		0.9949		
				B-MDM	74 ± 6	0.13		0.9950		
				OMC	83 ± 4	0.41		0.9913		
				IAMC	77 ± 5	1.04		0.9936		
				OCT	76 ± 5	1.38		0.9932		
				BP-3	93 ± 8	0.04		0.9924		
				EHS	63 ± 1	0.10		0.9924		
				BP-4	103 ± 4	0.03		0.9935		
				HMS	65 ± 3	0.11		0.9918		
				BP-1	106 ± 8	0.11		0.9937		
				BP-8	110 ± 6	0.03		0.9948		
	800 mL	Oasis HLB, adjusted to pH2-5.4 mL ethyl acetate and 3 mL methanol + evaporation time	GC–MS	BP	91 ± 4	1.6	5.3	0.999	NR	Kotnik et al. (2014)
				4-OHBP	95 ± 7	0.3	0.5	0.998		
				BP-1	93 ± 9	0.3	1.4	0.999		
				BP-3	95 ± 9	0.5	2.0	0.999		
				BP-8	91 ± 6	0.2	0.4	0.999		
	1 L	STRATA X, 59 mL methanol, adjusted to pH3, 9 mL ethyl acetate/ dichloromethane	UPLC-DAD	BP-3	94–104	1.4	4.8	0.9999	2–6	Sanchez Rodriguez et al. (2015)
				4-MBC	91–98	0.9	3.1	0.9999	2–7	
				OCT	80–100	2.8	9.3	0.9999	4–6	
				OMC	79–92	1.6	5.2	0.9999	6–14	
				OD-PABA	84–93	1.2	3.9	0.9999	6	
				HMS	78–110	2.4	8.0	0.9999	6–21	
				B-MDM	86–90	2.0	6.7	0.9995	5–8	
				DHHB	88–91	1.3	4.2	0.9998	6	
	100 mL	LC-18, 13 mL of methanol + evaporation time	UPLC-ESI–MS/MS	BP-1	92.2–96.9	3.68	11.15	0.9957	4–6.2	Li et al. (2017)
				BP-2	91.5–114.7	8.13	24.64	0.9987	5.7	
				BP-3	94.3–105.2	2.12	6.41	0.9968	3.3–6.6	
				BP-8	91.1–91.5	4.79	14.52	0.9970	3.5–4.1	
				OMC	91.6–114.4	3.25	9.85	0.9997	2.8–5.5	
				OCT	87.7–104.6	3.03	9.19	0.9988	1.6–4.3	
				4-MBC	85.3–110.3	2.59	7.84	0.9964	3.1–7.4	
				3-BC	87.6–98.3	8.70	26.38	0.9972	4.9–7	
				OD-PABA	101.3–111.2	4.91	14.88	0.9986	2.7–4.2	
				Et-PABA	110.2–110.6	9.17	27.79	0.9981	3.5–5.3	
				4-OHBP	91.6–96.3	8.76	26.56	0.9975	4.4–6.8	
				4-DHB	86.0–88.0	8.70	26.37	0.9961	4.6–5.9	
	350 mL	Bond Elut C18, 27 mL of 50:50 v/v methanol: ethyl acetate, 3.5 mL 5% (w/v) Na2EDTA + evaporation time	UPLC-ESI–MS/MS	BP-3	93 ± 8	0.04	NR	0.9924	8	Tsui et al. (2019)
				4-MBC	83 ± 4	0.28		0.9919	4	
				OCT	76 ± 5	1.38		0.9932	5	
				OD-PABA	73 ± 4	0.038		0.9927	4	
				B-MDM	74 ± 6	0.13		0.9950	6	
				OMC	83 ± 4	0.41		0.9960	4	
				BP-4	103 ± 4	0.03		0.9935	4	
				EHS	63 ± 1	0.1		0.9924	1	
				HMS	65 ± 3	0.11		0.9918	3	
				BP-1	106 ± 8	0.11		0.9937	8	
				BP-8	100 ± 6	0.03		0.9948	6	
HS-SPME	10 mL	35% NaCl, Na_2_S_2_O_3_	GC–MS/MS	EHS	84–112	0.69	NR	0.9977	6.4–9.1	Vila et al. (2016a)
				BS	92–123	0.12		0.9993	4–10	
				HMS	90–117	0.34		0.9987	5.1–12	
				IAMC	94–101	0.068		0.9996	5.7–10	
				BP-3	73–115	1.5		0.999	3.4–12	
				4-MBC	73–108	1.5		0.9996	5.2–8.7	
				ETO	60–119	1.5		0.9997	12–20	
				EHPABA	82–106	0.25		0.9997	6.8–7.6	
				OMC	75–117	0.22		0.9997	8.7–11	
				OCT	104–128	0.16		0.9967	2.9–15	
				B-MDM	NR	12		0.9964	7.4–11	
				DRT	94–121	3		0.9937	4.3–10	
				DHHB	89–99	6		0.9996	5.6–14	
DI-SPME	10 mL	0.1 g K_2_CO_3_, 0.2 mL acetic anhydride	GC–MS/MS	EHS	93.1–95.9	0.066	0.22	0.9938	− 6.3–9.7	Vila et al. (2017)
				IAMC	94.9–96.5	0.069	0.23	0.9970	3–5.6	
				HMS	85.6–90.8	0.15	0.49	0.9944	6–7.3	
				BS	91.4–97.7	0.084	0.28	0.9976	6.1–10	
				4-MBC	90.6–95.3	0.84	2.8	0.9981	6.5–18	
				BP3-4	86.9–95.3	0.3	1	0.997	9.6–11	
				Eto	87.5–106	0.15	0.51	0.9971	7.3–11	
				BP-1	92.8–103	6.1	20	0.9968	8.4–8.7	
				EHPABA	95.9–101	0.096	0.32	0.9991	7.3–8.3	
				OMC	86.8–98	0.06	0.2	0.9963	9–9.2	
				BP-8	79.9–102	8.2	27	0.9960	9–9.4	
				OCT	98.5–102	0.18	0.6	0.9967	4.2–5.5	
SBSE	100 mL	PDMS-coated stir bar, adjust to pH 2, 10% MeOH, extraction time 9 h	GC–MS	BP-3	27.6	2	NR	0.9971	1.3	Pintado–Herrera et al. (2013)
				OCT	59. 6	0.6		0.9981	7.8	
	100 mL	Acetic anhydride (500 mL), NaCl 100 g L^−1^, 10 g L^−1^ Na_2_CO_3_, 5 h agitation time	GC-APGC–ToF–MS	BS	50–100%	2.15	21.082	0.9402	NR	Pintado–Herrera et al. (2014)
				EHS		0.28	0.329	0.9482		
				HMS		0.44	0.335	0.9609		
				4-MBC		0.01	0.020	0.9763		
				OCT		0.02	0.029	0.9238		
				OMC		0.46	0.367	0.9738		
				OD-PABA		0.6	0.135	0.9690		
				2-OHBP		0.53	1.142	0.9632		
				B-MDM		12.4	28.986	0.9599		
				3-OHBP		0.61	0.857	0.9505		
				4-OHBP		1.55	0.857	0.9015		
				BP-3		0.17	0.921	0.9807		
				BP-10		1.66	0.393	0.9060		

^a^Liquid-liquid extraction (*LLE*), solid-phase extraction (*SPE*), direct immersion solid phase microextraction (*DI-SPME*), headspace solid phase microextraction (*HS-SPME*), stir bar sorptive extraction (*SBSE*), stir bar dispersive liquid microextraction (*SBDLME*)

^b^2,24,4-tetrahydroxybenzophenone (BP-2), 2-ethylhexyl salicylate (EHS), 2-hydroxybenzophenone (2-OHBP), 3-benzylidene camphor (3-BC), 3-hydroxyben-zophenone (3-OHBP), 4-(dimethylamino) benzoate (ethylhexyl dimethyl) PABA (EDP), 4,4-dihydroxybenzophenone (4-DHB), 4-hydroxybenzophenone (4-OHBP), 4-methylbenzylidene camphor (4-MBC), benzophenone (BP), benzophenone-1 (BP-1), benzophenone-10 (BP-10), benzophenone-4 (BP-4), benzyl salicylate (BS), butyl-methoxy-dibenzoylmethane (B-MDM), diethylamino hydroxybenzoyl hexyl benzoate (DHHB), dioxybenzone or benzophenone-8 (BP-8), drometrizole trisiloxane (DRT), ethyl-4-aminobenzoate (Et-PABA), ethylhexyl dimethyl PABA (EHPABA), etocrylene (ETO), homosalate (HMS), isoamyl p-methoxycinnamate (IAMC), octocrylene (OCT), octyl methoxycinnamate or 2-ethylhexyl-methoxycinnamate (OMC), octyl-dimethyl-p-aminobenzonic acid (OD-PABA), oxybenzone or benzophenone-3 (BP-3)

^*^NR not reported/found

Other important considerations are if derivatization and concentration steps are required, extraction time and ‘greenness’ of solvents used. In the compiled studies, the analytical instrument most often used is GC (Table 5). In our method, we employed LC since it has the advantage not requiring a derivatization step, which is required to increase chromatographic efficiency when using GC due to the high polarity of OUVFs (Jeon et al. 2006; Kotnik et al. 2014; Vila et al. 2017). The extraction technique most often used in the compiled studies and in earlier studies was SPE (Jeon et al. 2006; Bratkovics and Sapozhnikova 2011; Cadena-Aizaga et al. 2020). Compared to the developed SALLE method, SPE can require a large volume of sample and solvents and may require time consuming concentration and evaporation steps. SALLE was used in one other study compiled in Table 5 (Labille et al. 2020), and targeted four OUVFS, used 50 mL sample volume, 200 g/L Na_2_SO_4_, 5 mL methyl tertiary-butyl ether and required additional steps for concentration and evaporation. In contrast, our work used a smaller sample volume and did not require additional concentration and evaporation steps to achieve trace-level environmentally relevant MDLs and PQLs for the targeted OUVFs. Concentration by evaporation can increase sample processing time, reduce method robust, and can limit a methods potential for industrial scale processing. In summary, the developed SALLE method has advantages when compared with other methods regrading simplicity of operation, greenness of analytical method, and has a satisfactory performance suitable for its application.

### Environmental sample analysis

The optimised method was applied to the analysis of surface water samples collected at 19 sites in and near PPB, Australia. All environmental samples were spiked with surrogate oxybenzone-(phenyl-^13^C_6_) (100 ng/L), and surrogate recoveries were 77–151%. The sites represented a range of waterbody types including rivers near PPB, PPB beaches, the centre of the entry to PPB, and ocean beaches and rockpools near PPB (Fig. 1). Of the 7 target OUVFs, 4-MBC, B-MDM, OCT, and BP-3 were detected in one or more water samples collected at 10 of the 19 study sites (Table 6). Maximum total OUVF concentrations detected at sites ranged from 74 to 8597 ng/L per site, which were observed at Quarantine Station Beach and Williamstown Beach, respectively. The most widely detected OUVFs were 4-MBC (9 sites) and OCT (7 sites). Both BP-3 and B-MDM were detected at 4 sites. The most frequently detected OUVF was 4-MBC, which was detected in 37% of the water samples (29 samples), followed by OCT (18%, 14 samples), BP-3 (15%, 12 samples), and B-MDM (10%, 8 samples). B-MDM was detected at the highest maximum concentration (7968 ng/L), which was observed at Williamstown Beach, and was at least four-fold higher than the maximum concentration detected for 4-MBC (1643 ng/L, Sorrento Ocean Beach Rockpool), BP-3 (473 ng/L, Ricketts Point Beach), and OCT (99 ng/L, Sorrento Ocean Beach Rockpool).

**Table 6:** Maximum concentrations of the organic UV filters oxybenzone (BP-3), octocrylene (OCT), 4-methylbenzylidene camphor (4-MBC), octyl methoxycinnamate (OMC), butyl-methoxy-dibenzoylmethane (B-MDM), 2,4-dihydroxybenzophenone (BP-1), and 2,2′,4,4′-tetrahydroxybenzophenone (BP-2) detected in surface water samples collected at sites in and near Port Phillip Bay (PPB), Australia. Asterisk indicates concentration was below PQL, but above MDL, and a hyphen indicates not detected

Site	Activity level	Organic UV filter (ng/L)							Maximum total
		BP-2	4-MBC	B-MDM	OCT	OMC	BP-3	BP-1	
Rivers									
Maribyrnong River	None	-	-	-	-	-	-	-	-
Patterson River	None	-	-	-	-	-	-	-	-
Werribee River	None	-	-	-	-	-	-	-	-
Yarra River	None	-	-	-	-	-	-	-	-
PPB Beaches									
Brighton Beach	Medium	-	-	-	-	-	-	-	-
Carrum Beach	High	-	119*	-	-	-	-	-	119*
Geelong Eastern Beach	High	-	-	-	-	-	-	-	-
Mount Martha Beach	High	-	239	66	63	-	121	-	489
Portsea Pier	Low	-	-	-	-	-	-	-	-
Quarantine Station Beach	Low	-	-	-	74	-	-	-	74
Ricketts Point	Medium	-	207	-	-	-	473	-	680
Rye Pier	High	-	307	60	54	-	-	-	421
St Kilda Beach	Low	-	90*	-	-	-	-	-	90*
Williamstown Crystals	Low		108*	-	-	-	-	-	108*
Williamstown Beach	High	-	421	7968	70	-	138	-	8597
PPB Ocean Beaches									
Sorrento Ocean Beach	Medium	-	167	-	80	-	-	-	247
PPB Ocean Beach Rockpools									
Bridgewater Bay	Low	-	-	-	-	-	-	-	-
Sorrento Ocean Beach	High	-	1643	77	99	-	180	-	1999
PPB Entry									
Popes Eye	Low	-	-	-	80	-	-	-	80

The level of recreational activity was characterised for each site by counting the number of beachgoers at the site at the time of sampling since recreational activity has previously been shown as a significant source of OUVFs to marine environments (Labille et al. 2020). Comparing OUVF concentrations observed at sites characterised by high level of recreational activity (e.g. Rye Bay Beach, Carrum Beach, and Williamstown beach) with sites characterised by a low level of recreational activity (e.g. Quarantine Station Beach, St Kilda Beach, and Portsea Bay Beach) clearly showed that people engaged in recreational activities are associated with significant introduction of OUVFs to PPB (Table 6). Total maximum OUVF concentrations observed at sites characterised by a high level of recreational activity were 119–8597 ng/L, whereas total maximum OUVF concentrations observed at sites characterised by a low level of recreational activity were 74–108 ng/L. In addition, the profiles of OUVFs in surface water demonstrated site-specific differences (Table 6), possibly reflecting differences in the sunscreen products used by people engaged in recreational activities, the photostability of the OUVFs in seawater, and dilution of OUVFs by vertical and horizontal transport in the water column (Labille et al. 2020).

Three rivers were sampled during an ebbing tide to estimate the contribution that wastewater treatment plant effluent and urban runoff may have on concentrations of OUVFs occurring in PPB. In our study, OUVFs were not detected at river sites, which is interesting since common OUVF have been previously detected in PPB estuaries (Allinson et al. 2018). Allinson et al. (2018) reported concentrations of 4-MBC, OMC, and OCT up to 642 ng/L, 640 ng/L, and 109 ng/L, respectively, and the average OUVF concentrations across sites were 7.4 ng/L. Differences between our study and Allinson et al. (2018) with regards to sampling time and location may have resulted in OUVFs not being detected in our study since OUVFs can be subject to photodegradation and dilution by transport in the environment.

Temporal variation in total OUVF concentration was studied at two sites, Rye Bay Beach, and Sorrento Ocean Beach rockpool (Fig. 1). Samples were collected in the early morning (low recreational activity), at midday (high recreational activity), and in the early evening (low recreational activity). Sampling times coincided with different tidal stages (supplementary material, Table S1). The geomorphology of the sites differed; Sorrento Ocean Beach rockpool represents a closed system that is flushed at hightide, whereas Rye Bay Beach is a sandy embayment within PPB subject to continual tidal movements and circulating Bay currents. At both sites, the highest total OUVF concentration was observed at midday during peak recreational activity and was 795 ng/L at Rye Bay Beach and 4716 ng/L at Sorrento Ocean Beach rockpool. In the evening, OUVFs were observed at both sites; however, the total OUVF concentrations were lower than at midday and were 536 ng/L and 516 ng/L at Sorrento Ocean Beach rockpool and Rye Ocean Beach, respectively. In addition, OUVF profiles at the sites differed between midday and evening. At both sites, B-MDM, OCT, BP-3, and 4-MBC were detected at midday, but in the evening, only 4-MBC was detected at Rye Ocean Beach, and OCT, BP-3, and 4-MBC were detected in the evening at Sorrento Ocean Beach rockpool.

Unexpectedly, OCT was detected at Popes Eye (80 ng/L, no recreational activity) and Quarantine Station Beach (74 ng/L, low level of recreational activity) (Table 6). Both sites are close the entrance to PPB and are subjected to high tidal movement of water caused by the narrow entry to the bay. In similar studies in marine environments, detected OCT concentrations have ranged from 75 ng/L to 171 µg/L (Tsui et al. 2014; Vila et al. 2016b). Unlike other OUVFs included in this study (e.g. B-MDM and BP-3), which were not detected at these sites, OCT is photostable and may be more likely transported vertically and horizontally in the waterbody (Santos et al. 2012; Manasfi et al. 2017; Labille et al. 2020).

The concentration values detected at beaches are similar to those reported in many other studies in coastal areas. For instance, Labille et al. (2020) surveyed Mediterranean beaches and found that OCT and B-MDM were most frequently detected with concentrations ranging from 75–425 ng/L to 10–350 ng/L, respectively, whereas BP-3 and OMC were detected less often with concentrations ranging from 50–75 mg/L to 2.6–8.8 ng/L, respectively. In coastal areas, OUVFs are typically detected at trace levels. For some OUVFs, however, higher maximum concentrations have been observed, for example BP-3 has been detected at 1.395 mg/L (Downs et al. 2016), OCT at 79 µg/L (Vila et al. 2017), and B-MDM at 72 µg/L (Vila et al. 2016a).

The differences in OUVF concentrations and profiles observed among sites in our study show that OUVF input to PPB is due to recreational activities and that the OUVF input is pulsed based on the activity level of beachgoers. This was particularly evident in the temporal study at Sorrento Ocean Beach rockpool. The site was sampled in the early morning (low recreational activity, no OUVFs detected), at midday before the pool was reached by the flooding tide, and in the evening after the pool had been flushed by the high tide with similar OUVF profiles observed at both times, but a higher total concentration observed at midday corresponding to peak recreational activity. Furthermore, the temporal study at Rye Bay Beach showed how OUVF environmental concentrations are reduced by dilution occurring due to transport in the water column and/or waterbody influenced by currents and site geomorphology.

Presently, there are no marine water quality guideline values for the target OUVFs in Australia (ANZECC & ARMCANZ 2000, 2018). Predicted no-effect concentrations (PNECs), which are important indicators of ecological risk, for the detected OUVFs have been reported as ≥ 40 ng/L 4-MBC, ≥ 100 ng/L OCT, and ≥ 10 ng/L BP-3 (Carve et al. 2021; Miller et al. 2021), but for B-MDM, no PNECs are available due to insufficient ecotoxicological data being available for their calculation. In this context, PNECs were exceeded at 9 sites for 4-MBC and 4 sites for BP-3. Furthermore, it is important to consider that OUVF concentrations can be higher in biota than observed in water samples due to bioconcentration (Cadena-Aizaga et al. 2022), and that the toxic effects of OUVF mixtures can occur at concentrations lower than observed for a single chemical (Escher and Hermens 2002). To this end, of the four OUVFs detected in this study, two or more were detected in 15 water samples, and in particular, OCT’s high log K_ow_ (> 6) indicates its potential for bioaccumulation (Cadena-Aizaga et al. 2022). Results from this study indicate that the potential risk posed by 4-MBC, OCT, and BP-3 to Port Philip Bay aquatic ecosystems are appreciable, and further assessments of their occurrence in PPB and associated biota are paramount to evaluating their ecological risk.

## Conclusion

A sensitive analytical method based on salting-out assisted liquid–liquid extraction (SALLE) and LC–MS/MS method has been developed. The method enables the determination of seven OUVFs at trace level in environmental water samples with good trueness and precision. The method is appropriate for analysis of target compounds at trace concentrations with low relative standard deviation (< 6%) and limits of detection (MDLs: 11 to 45 ng/L and PQLs: 33 to 135 ng/L). The proposed method is simple and efficient, and the protocol uses minimal amounts of organic solvents (10 mL/sample) and time (< 1.5 h/sample).

To our knowledge, this is the first report of the occurrence of the target OUVFs in the temperate environments in and near Port Phillip Bay, Australia. Results indicate that 4-MBC, BP-3, B-MDM, and OCT are detectable in the coastal surface water of Port Phillip Bay during summer and may pose an ecological risk to PPB. The OUVF B-MDM was detected at the highest concentrations, and 4-MBC was detected most widely. Williamstown Beach ranked the highest in terms of the total OUVF concentration present at a site. The concentrations of OUVFs detected at sites reflected the level of recreational activity observed at the time of sampling.

The temporal changes in OUVF concentration reflected patterns of recreational activities and localised water movement, influenced by tidal cycles, currents, and site geomorphology. Results suggest that OUVF input to the PPB marine environments is pulsed due to the activity of beachgoers, and that the persistence of OUVFs is influenced by water residence time at the site and OUVF stability in the environment. These data presented are essential to evaluating the potential risk posed by OUVFs to the Port Philp Bay marine environment.

### Supplementary Information

Below is the link to the electronic supplementary material.

Supplementary file1 (DOCX 85.1 KB)

## Data Availability

The data presented in this study are available on request from the corresponding author.
