# Supplementary material for: Salting-out assisted liquid–liquid extraction combined with LC–MS/MS for the simultaneous determination of seven organic UV filters in environmental water samples: method development and application
Source: Environ Sci Pollut Res Int. 2023 Sep 15;30(47):104870–85. doi: 10.1007/s11356-023-29646-8 (PMC10567945; doi:10.1007/s11356-023-29646-8)
Supplement: Supplementary file 1 — Supplementary file1 (DOCX 85.1 KB) [file 11356_2023_29646_MOESM1_ESM.docx]

Supplementary File

For each site, the number of people engaged in recreational activity was estimated by counting the number of beachgoers in the water. Climate data on daily solar exposure (MJ m^−2^) and weather parameters (temperature, wind speed and tide state) was compiled using publicly available data on the Australian Bureau of Meteorology website for the nearest observation station (Australian Bureau of Meteorology, 2022). Water and air temperature were collected at the site. Air temperature during midday on the days of sampling was ~ 30 ℃. Wind strength was gentle and < 20 km/h. Daily global solar exposure was ~30 MJ m^−2^. For each site, sampling date and time, and water temperature and tide stage during sampling are shown in Table S1.

**Table S1**. Summary of site environmental data and density of people engaged in recreational activities (number of people/10 m^2^).

| Site | Date | Time | Water Temp (℃) | Tide stage |
| --- | --- | --- | --- | --- |
| Patterson River | Jan 22, 2022 | 16:25 | 23.6 | Flood (13:00 low, 18:37 high) |
| Yarra River | Jan 22, 2022 | 11:53 | 16.9 | Ebb (7:23 high, 13:29 low) |
| Werribee River | Jan 24, 2022 | 12:00 | 23.8 | Ebb (8:00 high, 14:25 low) |
| Maribyrnong River | Jan 22, 2022 | 11:30 | 24 | Ebb (7:26 high, 13:33 low) |
| Carrum Beach | Jan 22, 2022 | 16:55 | 26.2 | Flood (13:00 low, 18:37 high) |
| Ricketts Point Beach | Jan 22, 2022 | 15:40 | 27.5 | Flood (13:00 low, 18:37 high) |
| Quarantine Station Beach | Jan 23, 2022 | 13:15 | 18.2 | Flood (10:25 low, 16:35 high) |
| Rye Bay Beach (AM) | Jan 23, 2022 | 8:10 | 17.9 | Ebb (7:06 high, 13:17 low) |
| Rye Bay Beach (MD) | Jan 23, 2022 | 14:30 | 22.4 | Flood (13:17 low, 18:53 high) |
| Rye Bay Beach (PM) | Jan 23, 2022 | 20:10 | 14.1 | Ebb (18:53 high, 1:10 low) |
| Portsea Bay Beach | Jan 23, 2022 | 12:45 | 17.8 | Flood (11:51 low, 17:42 high) |
| Williamstown Beach | Jan 22, 2022 | 12:30 | 23.6 | Ebb (7:19 high, 13:27 low) |
| Williamstown Crystals | Jan 22, 2022 | 12:45 | 23.6 | Ebb (7:19 high, 13:27 low) |
| Brighton Beach | Jan 22, 2022 | 14:50 | 24.6 | Flood (13:03 low, 18:41 high) |
| Mount Martha Beach | Jan 23, 2022 | 17:00 | 22.8 | Flood (13:44 low, 19:22 high) |
| Geelong Eastern Beach | Jan 24, 2022 | 13:30 | 26.7 | Ebb (8:40 high, 15:04 low) |
| St Kilda Beach | Jan 22, 2022 | 14:00 | 25.2 | Flood (13:23 low, 18:40 high) |
| Sorrento Ocean Beach | Jan 23, 2022 | 12:15 | 19.2 | Flood (10:24 low, 4:34 high) |
| Sorrento Rockpool (AM) | Jan 23, 2022 | 8.50 | 15.6 | Ebb (4:36 high, 10:24 low) |
| Sorrento Rockpool (MD) | Jan 23, 2022 | 12:15 | 19.2 | Flood (10:24 low, 4:34 high) |
| Sorrento Rockpool (PM) | Jan 23, 2022 | 20:40 | 21.6 | Ebb (4:34 high, 22:18 low) |
| Bridgewater Bay Rockpool | Jan 23, 2022 | 9:30 | 18.1 | Ebb (4:11 high, 10:52 low) |
| Popes Eye | Jan 21, 2022 | 11:00 | 17 | Flood (9:14 low,15:11 high) |


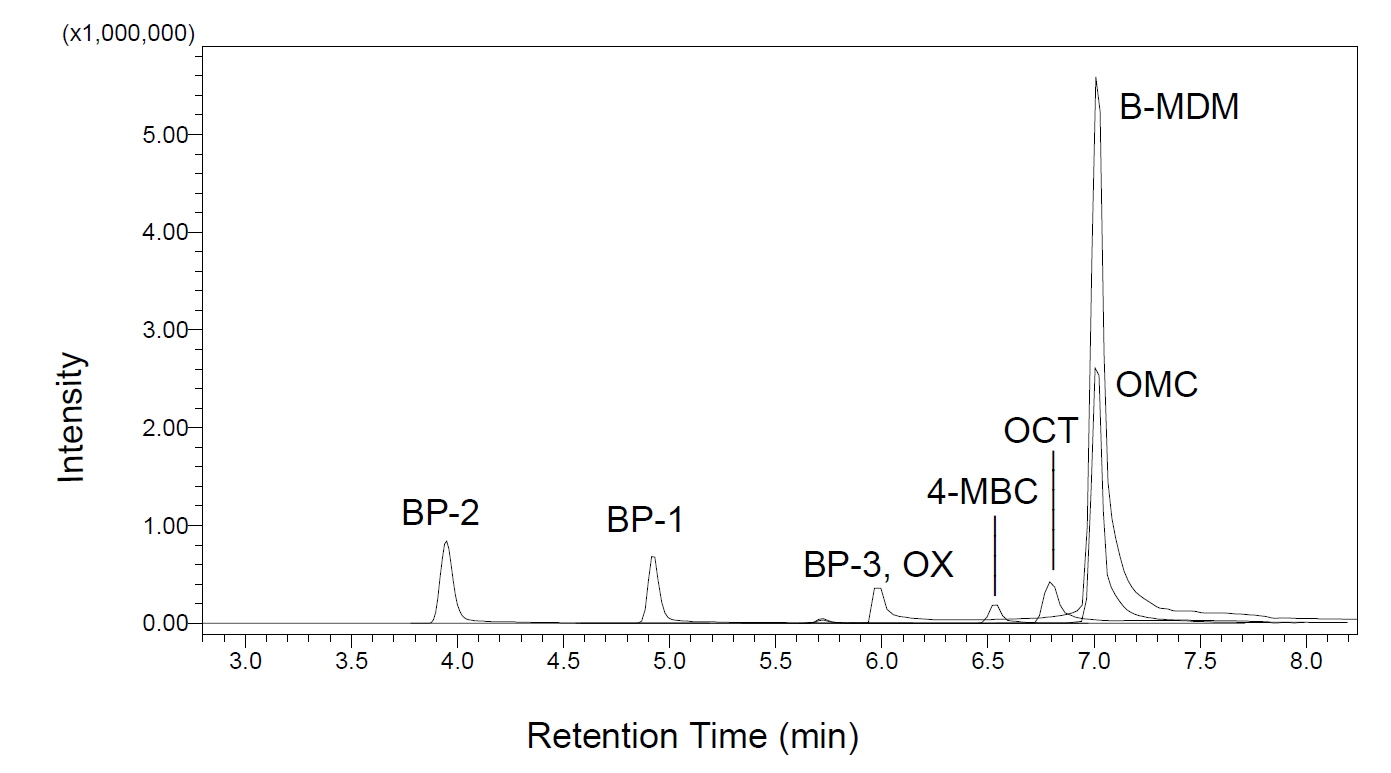


Figure S1. A representative chromatogram for the organic UV filter combined standards solution (2,4-dihydroxybenzophenone (BP-1), 2,2',4,4'-tetrahydroxybenzophenone (BP-2), oxybenzone (BP-3), 4-methylbenzylidene camphor (4-MBC), butyl-methoxy-dibenzoylmethane (B-MDM), octyl methoxycinnamate (OMC), and octocrylene (OCT)) and the isotopically labelled analogue oxybenzone-(phenyl-^13^C_6_) (OX).
